# Neuromyelitis Optica Spectrum Disorder: A Rare Cause of Spinal Cord and Optic Nerve Involvement

**DOI:** 10.7759/cureus.23641

**Published:** 2022-03-30

**Authors:** Steven Douedi, Lauren Pilarz, Hala Al Kallas, Viraaj S Pannu, Mihir Odak, Ann Kozlik, Sarah Elmedani, Islam Elkherpitawy, Natasha Campbell

**Affiliations:** 1 Internal Medicine, Jersey Shore University Medical Center, Neptune City, USA; 2 Internal Medicine, St. George's University School of Medicine, True Blue, GRD

**Keywords:** demyelinating neurological disorder, demyelinating, transverse myelitis, optic neuritis, autoimmune disease, neuromyelitis optica spectrum disorder

## Abstract

Neuromyelitis optica spectrum disorder (NMOSD) is defined as a rare central nervous system, demyelinating, autoimmune disorder with autoantibodies against aquaporin-4. Commonly affecting females, NMOSD is known to also be a relapsing disease and can increase in severity during each episode. Diagnostic criteria include ruling out multiple sclerosis, spinal magnetic resonance imaging, and autoantibody detection. While management focuses on relapse treatment and prevention, high-dose steroids and plasma exchange have proven to be useful options. We present a case of a young female presenting with NMOSD relapse appropriately managed with plasma exchange.

## Introduction

Neuromyelitis optica spectrum disorder (NMOSD) is defined as a rare central nervous system, demyelinating, autoimmune disorder with autoantibodies against aquaporin-4 [1]. NMOSD is usually limited to the spinal cord and optic nerve manifested as transverse myelitis and optic neuritis [1]. The general onset of NMOSD is predominantly in the female population with a female to male ratio of 9:1, the median age of 40, and non-Caucasian race [1-3]. It has also been suspected that females have more relapses, each becoming more severe and frequent compared to male counterparts; however, further studies are required to confirm this association [2,3]. We present a case of a 38-year-old Hispanic female with a history of NMOSD who presented with relapsing disease found on imaging and required aggressive treatment.

## Case presentation

A 38-year-old Hispanic female with a medical history of NMOSD for one year (diagnosed with imaging and NMO-IgG antibody positivity) ambulates with a walker, due to worsening weakness over the last three months and a cerebral aneurysm status post-clipping, and presents to the emergency department with a five-day history of sharp headaches located in the back of her head, worsening weakness of her left arm especially at night, urinary retention, constipation, and intermittent left ear pain for a week. The patient admits to developing shingles over the past week under her right breast at another facility, treated with 1 gram valacyclovir but denies right arm weakness or numbness. Since her diagnosis of NMOSD, she has been treated with prednisone 20 milligrams (mg) orally (PO) daily, mycophenolate mofetil 250 mg PO twice daily, baclofen 10 mg PO three times daily, gabapentin 300 mg PO three times daily, and duloxetine 30 mg PO daily. She has also received multiple plasma exchange treatments, the last of which happened six months prior to this admission, due to hospitalization with a complaint of similar weakness. The plasma exchange treatments are usually helpful for two to three months after which the effect wears off and her symptoms relapse. 

**Figure 1:**
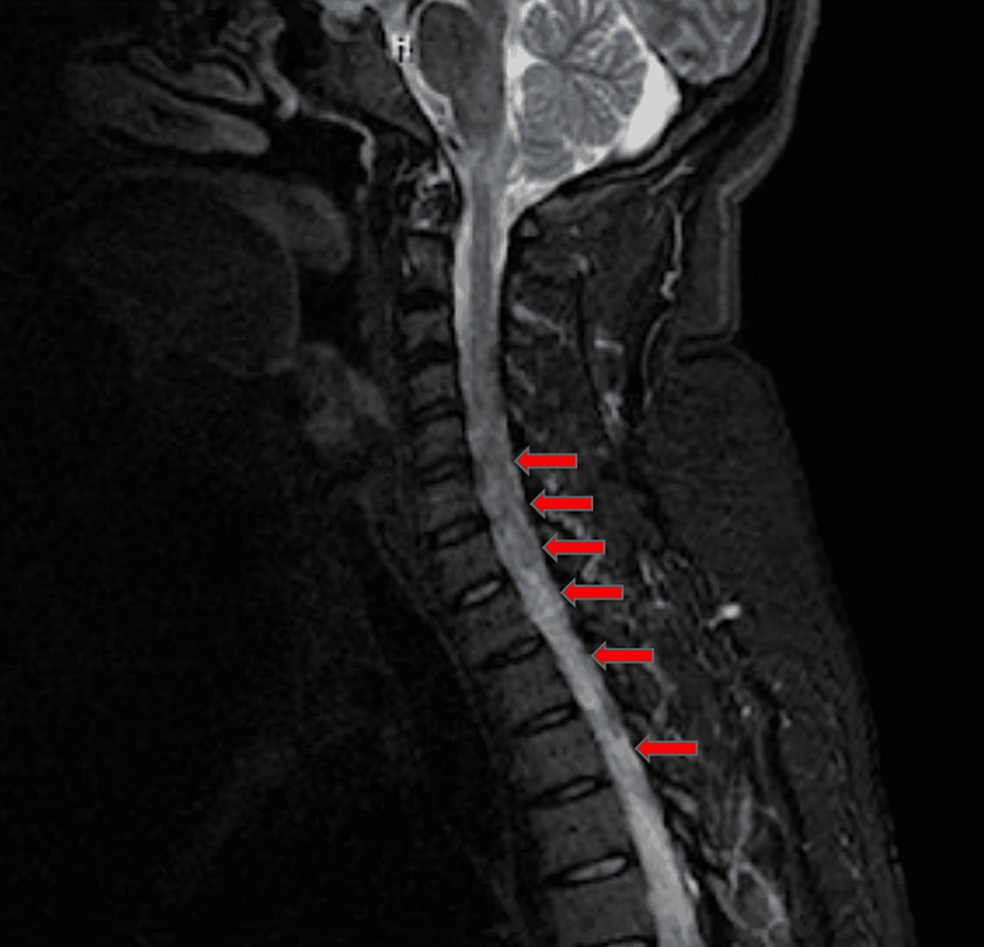
Magnetic resonance imaging of the cervical spine showing signal abnormalities of the cord involving the central aspect with inflammatory etiologies consistent with neuromyelitis optica (red arrows).

**Figure 2:**
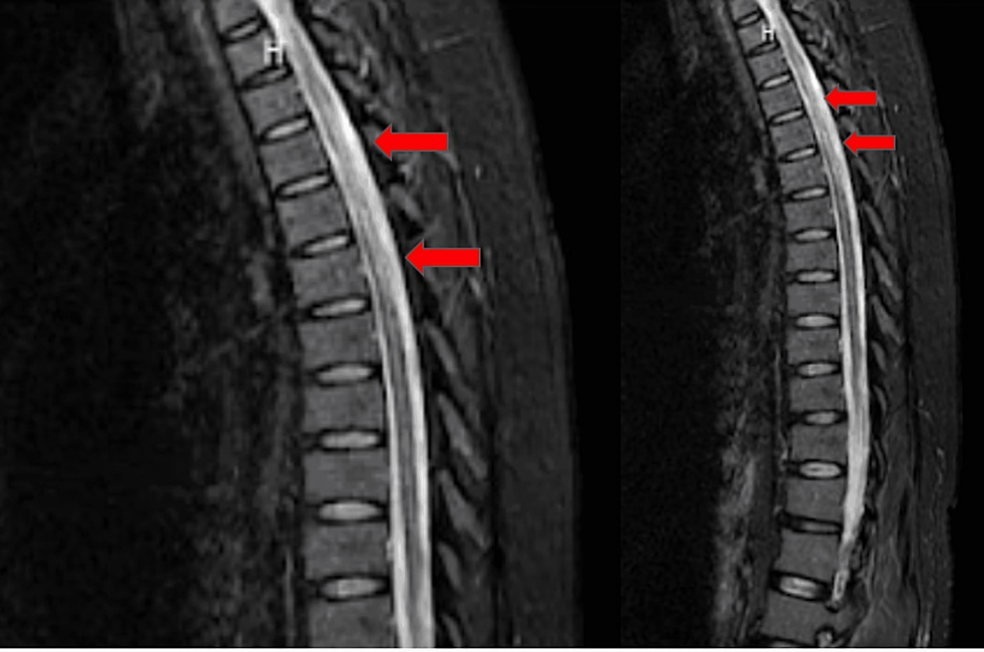
Magnetic resonance imaging of the thoracic spine showing signal abnormalities of the cord involving the central aspect with inflammatory etiologies consistent with neuromyelitis optica (red arrows).

Upon physical examination, the patient was unable to lift her bilateral legs, with strength quantified at 0/5, and had upper extremity weakness, especially on the left side, with strength quantified as 2/5. Cranial nerves were intact and sensation to light touch was decreased in the bilateral lower extremities. The patient had mild tenderness generalized to the abdomen. A crusted rash was appreciated underlying the right breast with a T5 dermatomal distribution, wrapping around to the back that does not cross the midline. Laboratory findings on admission were significant for a low hemoglobin (10.2 g/dL; reference range: 12 - 16 g/dL), low hematocrit (33.4%, reference range: 35-48%), MCV (80.1), and high platelet count (588k uL, reference range: 150 - 450k uL) but were otherwise unremarkable. A computed tomography (CT) scan of the head was unremarkable and a CT angiogram of the head and neck was performed, which showed no evidence of stenosis or occlusion. Magnetic resonance imaging (MRI) of the cervical and thoracic spine showed signal abnormalities of the cord involving the central aspect with inflammatory etiologies consistent with NMOSD (Figure 1 and Figure 2). MRI of the brain was also performed but was unremarkable. 

Her home medications were continued and neurology was consulted for plasmapheresis treatment, which was initiated on day three of admission and planned for a total of five treatments. She ultimately completed a 10-day course of valacyclovir for shingles with cephalexin and nystatin cream added as prophylaxis to the affected areas. Following two plasmapheresis treatments, the patient was able to flex the fingers of her left hand and had 2/5 strength in her left proximal arm. She continued to have weakness in her distal left arm and the patient was still not able to move her lower extremities on command. Following the fourth plasmapheresis treatment, her strength in the left upper extremity increased to 4/5 overall and 0/5 in the lower extremities bilaterally. However, the patient flexed her legs when asked to raise her upper extremities. Following the patient’s fifth plasma exchange treatment, the strength in her left upper extremity was stable at 4/5 and the strength in her lower extremities gradually improved from 0/5 to 1/5 one day after the fifth treatment, to 2/5 two days after, and 3/5 five days after completing treatment. She was ultimately discharged in stable condition to undergo rehab for continued strength training and physical therapy with plans for neurology outpatient follow-up.

## Discussion

NMOSD is a demyelinating autoimmune central nervous system disorder limited to the spinal cord and optic nerve [1]. Commonly affecting females, NMOSD is known to also be a relapsing disease and can increase in severity during each episode [1]. Recent studies and findings have separated NMOSD from being a subset of multiple sclerosis (MS), a favorable differential diagnosis, and have found a strong association with the autoantibodies to aquaporin-4, which has also been found to correlate with the severity of the disease [1,4]. The diagnosis of NMOSD requires two of the following three: brain MRI ruling out MS, spinal MRI showing three or more contiguous vertebral segments with lesions, and NMO-IgG seropositivity [1]. Our patient in this case report met all three criteria on a previous admission and had a confirmed diagnosis of NMOSD prior to her presentation. Cerebrospinal fluid analysis (CSF) of NMOSD also differentiates the disease from MS. In recent studies, only 18% of patients with confirmed NMOSD had oligoclonal bands in CSF analysis, and may not be found at all in patients in remission [1,5]. On brain MRI, lesions in NMOSD patients are primarily seen around aquaporin-4 expression areas such as the diencephalon compared with MS findings [5,6,7]. 

Despite several advancements in research on NMOSD patients, treatment has still yet to be defined. Management primarily focuses on relapses and preventing recurrent events [1]. Typically patients are treated with high-dose intravenous steroids during relapse attacks and should be considered for plasma exchange [1,8]. In our patient, given plasma exchange in the past had improved release symptoms, she was started on similar treatment as initial management [8]. If both high-dose intravenous steroids and plasma exchange fail to improve symptoms, intravenous immunoglobulins have shown benefits in NMOSD patients [1,9]. Patients with recurrent relapses should also be considered for chronic immunosuppressive therapy. Studied therapies for NMOSD immunosuppressive therapy include rituximab and azathioprine and prednisone, although further research is needed for long-term management [10,11].

## Conclusions

NMOSD, although uncommon, is a debilitating, demyelinating autoimmune central nervous system disease that clinicians should be aware of in the younger female population. Although there are several similarities to the presentation and treatment of multiple sclerosis, NMOSD is difficult to manage and presents more often as a relapsing disease with no known cure as yet. Our patient, with a known diagnosis of NMOSD, was treated successfully with plasma exchange for her relapse as an initial treatment given her previous success. While long-term immunosuppressive therapy has shown benefits in the prevention of relapse in literature, physical therapy and rehabilitation should also be included in the management of these patients. 
